# Prevalence and Correlates of Musculoskeletal Pain in Adults with Type 2 Diabetes in Populations with Low-Risk of Obesity: A Cross-Sectional Study

**DOI:** 10.4314/ejhs.v30i6.13

**Published:** 2020-11

**Authors:** Adedapo W Awotidebe, Auwalu Shehu

**Affiliations:** 1 Department of Physiotherapy, Faculty of Allied Health Sciences, Bayero University Kano, P.M.B. 3011, Kano State, Nigeria; 2 Department of Physiotherapy, Aminu Kano Teaching Hospital, P.M.B. 3452, Kano State, Nigeria

**Keywords:** Prevalence, Musculoskeletal Pain, Type 2 Diabetes, Obesity, Physical Activity

## Abstract

**Background:**

There are few data concerning the prevalence and predictors of musculoskeletal pain among adults with type 2 diabetes in population with low-risk of obesity. Our objective was to describe the point prevalence and factors associated with increased risk of musculoskeletal pain in this population.

**Methods:**

A cross-sectional data of 200 adults with type 2 diabetes, aged ≥ 18 years who were attending two tertiary hospitals were examined. Musculoskeletal pain and physical activity were collected with Nordic Musculoskeletal Questionnaire (NMQ) and International Physical Activity Questionnaire (IPAQ-SF) respectively. We used logistic regression to examine the risks associated with musculoskeletal pain.

**Results:**

The point prevalence of musculoskeletal pain was 72.7% and similar between men (72.3%) and women (73.1%). In the last 7days, advancing age (odds ratio=1.09;95%CI:1.02–1.16) and comorbidity (odds ratio=3.0;95%CI:1.07–8.39) were risk factors associated with musculoskeletal pain. In the last 12 months, only comorbidity (odds ratio=5.57;95%CI:1.62–19.17) was a risk factor for increasing musculoskeletal pain. However, a unit increase in physical activity level (odds ratio=0.06;95%CI:0.008–0.51) was associated with decreased odds of musculoskeletal pain.

**Conclusions:**

The prevalence of musculoskeletal pain was high and physical activity was associated with a decreased risk thereof. A further research should be evaluated on the influence of physical activity on musculoskeletal pain.

## Introduction

Type 2 diabetes mellitus is one of the leading causes of mortality globally (1), a development that has contributed to the socio-economic burden and unfavourable economic indices in both developed and developing nations (2). People with type 2 diabetes have a higher prevalence of musculoskeletal pain and its manifestation in this population accounts for a marked increase in institutionalization and hospital visits (3). In two cross-sectional studies, the prevalence of musculoskeletal pain in adults above 18 years with type 2 diabetes ranged from 46.5% to 82% (4,5). The prevalence of musculoskeletal pain increases with age and is higher in women (6). The mechanism of musculoskeletal pain in adults with type 2 diabetes is less clear; however, several factors have been observed to exacerbate the propensity for musculoskeletal pain. These include neuropathy (7) vascular insufficiencies (8), vitamin D deficiency (9), decreased insulin-like growth factors (IGF-1) (10), sedentary behaviours (11), reduced dietary intake (12) and obesity (13). Musculoskeletal pain is associated with decreased physical function and quality of life (6). Therefore, improving the physical activity (PA) level of patients with type 2 diabetes is crucial to promoting physical function and quality of life. Guidelines for physical activity in this population recommend moderate-intensity exercise of at least 150 minutes per week. This threshold is associated with improved glycaemic control and insulin sensitivity (14). Apart from aerobic exercise, it has also been observed that resistance training reduces the prevalence of hyperglycaemia in patients with type 2 diabetes (14).

However, there are concerns that inherited genetic susceptibility, behavioural risk variations (e.g. obesity and sedentary lifestyle), and ethnic differences are central to defective insulin secretion and insulin resistance (15). This phenomenon underscores the variations in vascular complications and risk factors associated with type 2 diabetes (16). For example, the effect of environmental factors (obesity) and difference in genetic origin are some of the reasons why the prevalence of type 2 diabetes is low in African countries compared with Western countries (17). Consequently, there is a paucity of studies in African populations on the prevalence of musculoskeletal pain and its risk factors in populations with low risk of obesity. Studies in African populations are thus needed. The aim of this study therefore was to determine the point prevalence of musculoskeletal pain and its risk factors in adults with type 2 diabetes in Northern Nigeria. We hypothesized that the prevalence of musculoskeletal pain in this setting will be lower than what was reported elsewhere.

## Materials and Methods

This was a cross-sectional hospital-based study was conducted on adults with type 2 diabetes, aged 18 or older who were attending the endocrinology clinic of two tertiary hospitals in Kano metropolis, Nigeria. We used the diabetes registers, which included information on diagnosis, co-existing conditions, treatment and demographic characteristics, to recruit participants into the study. The participants were recruited consecutively during routine treatment visits. However, patients diagnosed with type 2 diabetes but either had a physical disability and/or a diabetic foot were excluded from the study.

The sample size for this study was estimated using single proportion formula (18) taking previous prevalence of musculoskeletal pain in this population, 82.6% (5) and a precision error of 5%, we used a priori sample size parameters (Z = 1.96; P=0.826; d=0.05) to estimate an appropriate sample size of 220.

**Data collection, variables and sources of data**: Data were collected between August 01 and November 31, 2017. Demographic variables obtained include age, gender, level of education, disease duration and co-existing conditions. Body weight and height were measured according to the standards of the International Society for the Advancement of Kinanthropometry (ISAK) (19). We collapsed both “Obesity” (BMI ≥ 30 kg/m^2^) and “Overweight” (BMI 25–29.99 kg/m^2^) into one category “Obesity” in order not to violate the assumptions of chi-square concerning the minimum expected cell frequency. Underweight was defined as BMI < 18.5 kg/m^2^ and “Normal” weight as BMI of 18.5–24.99 kg/m^2^. The Standardized Nordic Musculoskeletal Questionnaire (20) was used to assess the presence of musculoskeletal pain in nine major body regions (neck, shoulders, upper back, lower back, elbows, wrists, thighs, knees and ankles) in the 7 days (acute pain) and 12 months (chronic pain) before the study. The presence of musculoskeletal pain in at least one body region was categorized as (Yes pain: 1) and no pain in any body region was categorized as 0 (No pain: 0). The dichotomous variable (Yes pain: 1; No pain: 0) was used to estimate point prevalence of musculoskeletal pain in the last 12 months. The International Physical Activity Questionnaire-short version (IPAQ-Short Form) (21) was used to assess the frequency, intensity, duration and type of seven-day recall of total physical activity. The total PA was defined as “Low PA” (< 3 MET, Metabolic Equivalent Task), “Moderate PA” (3–6 MET) and “Vigorous PA” (> 6 MET) (22). One MET is defined as the energy expended (or amount of oxygen consumed) at rest and is roughly equal to 3.5 ml of O_2_ per kilogram body weight multiplied by a minute in adults.

**Ethical approval**: Ethical approval was obtained from the Aminu Kano Teaching Hospital Research Ethics Committee (AKTH/MAC/SUB/12A/P-3/VI/2110) and the ethical protocols of the Declaration of Helsinki (1967) including the ethical principles of informed consent, voluntary participation and withdrawal, privacy and confidentiality, were followed to ensure wellbeing, safety and protection of study participants (23).

**Data analysis and statistics**: Descriptive statistical techniques in the form of tables, charts and percentages were used to present the prevalence and patterns of musculoskeletal pain in the last 12 months. Inferential statistics of Chi-square test (χ^2^) for independence (with Yates Continuity Correction) were used to determine the association between the presence of musculoskeletal pain (1= Yes; 0 =No pain) and participants' characteristics (sex, age, education, presence of co-existing condition, BMI, and PA). Multivariate logistic regression (stepwise forward selection method) was used to determine the contribution of each of age, sex, education, co-morbidity, disease duration, body mass index and physical activity to the presence of musculoskeletal pain. Assumptions for logistic regression were checked for multicollinerity (Cook's distance), model's fit (Omnibus test; Hosmer and Lemeshow test), and potential outliers were checked. The data were analysed using the Statistical Package for Social Science (SPSS) version 22 and significance level (alpha level) was set at 0.05.

## Results

**Characteristics of the study population**: A total of 198 adults with type 2 diabetes, with a mean age of 48.3 years were examined (Table 1). The majority of the participants were females (52.5%), had informal education (47%) and had comorbidity (40.9%). Their mean disease duration was 3.24 ± SD 2.78 years; mean BMI was 25.85 ± SD 4.09 kg/m^2^ and mean total physical activity was 6.81 ± 0.93 MET. Overall, 42.9% had low physical activity.

**Table 1:** Demographic and clinical characteristics and outcomes of adults with type 2 diabetes in low risk obesity populations

Variables	All (n=198)	Without MSP (n=54)	With MSP (n=144)	P-value
**Age, year**	48.26±10.73	46.19±10.62	53.58±12.31	0.001
**Sex, n (%)**				
**Male**	94(47.5)	26(48.1)	68(47.2)	0.907
**Female**	104(52.5)	28(51.9)	76(52.8)	
**Education, n (%)**				
**Informal**	93(47.0)	9(16.7)	84(58.3)	
**Primary**	27(13.6)	9(16.7)	18(12.5)	0.001
**Secondary**	24(12.1)	9(16.7)	15(10.4)	
**Tertiary**	54(27.3)	27(49.9)	27(18.8)	
**Comorbidity., n (%)**				
**No**	117(59.1)	44(81.5)	73(50.7)	0.001
**Yes**	81(40.9)	10(18.5)	71(49.3)	
**PA (%)**				
**Low**	85(42.9)	10(18.5)	75(52.1)	0.001
**Moderate**	103(52)	38(70.4)	65(45.10	
**High**	10(5.1)	6(11.1)	4(2.8)	
**Duration, year**	3.24±2.78	2.88±3.10	4.69±3.62	0.001
**BMI (kg/m^2^)**	25.85±4.09	25.66±3.45	25.59±4.07	0.910
**Moderate PA (MET)**	5.35±1.08	5.73±0.97	5.12±1.10	0.001
**Vigorous PA (MET)**	6.00±1.14	6.58±0.86	5.75 ±1.17	0.001
**Total PA (MET)**	6.81±0.93	7.09±1.14	6.05±1.30	0.001

**Figure 1:**
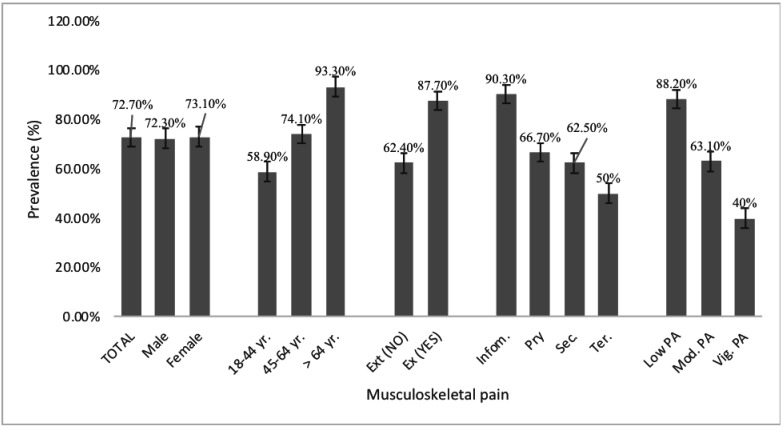
Prevalence of musculoskeletal pain in adults with type 2 diabetes according to gender, age, existing conditions, education and physical activity level

**Figure 2:**
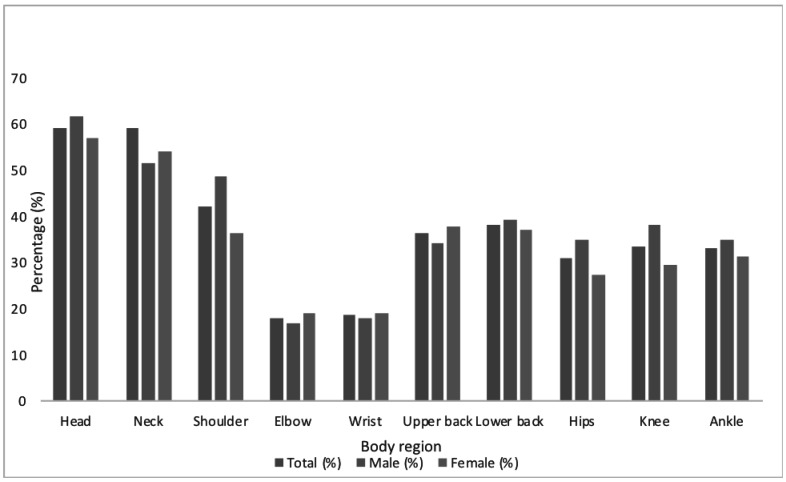
Pattern of musculoskeletal pain in adults with type 2 diabetes in a low-risk obesity population.

**Prevalence and pattern of musculoskeletal pain**: The overall prevalence of musculoskeletal pain was 72.7%, similar in men and women. (Figure 1). Prevalence of musculoskeletal pain increased with age, comorbidity, low education and sedentary behaviour (Figure 1). Most occurrences of musculoskeletal pain were in the upper extremities, in the head (59.3%), neck (59.3%) and shoulder (42.4%) (Figure 2). The prevalence of musculoskeletal pain was least in the wrist (18.6%) and the elbow (18.1%).

**Risk factors for musculoskeletal pain**: Chi-square test (χ^2^) of independence indicated a significant association between musculoskeletal pain status and age [seven-day MSP: χ^2^ (2) = 30.70, p<0.001; 12-month MSP:χ^2^ (2) = 11.91, p<0.05], education [seven-day MSP:χ^2^ (3) = 48.71, p<0.001; 12-month MSP: χ^2^ (2) = 30.34, p<0.001] and comorbidity status [seven-day MSP: χ^2^ (1) = 30.75, p<0.001; 12-month:χ^2^ (2) = 15.40, p<0.001] (Table 2). Similarly, results suggested a significant association between musculoskeletal pain and physical activity status [seven-day MSP: χ^2^ (2) = 32.07, p<0.001; 12-month MSP: χ^2^ (2) = 20.53, p<0.001].

**Table 2:** Association of selected factors with musculoskeletal pain of adults with type 2 diabetes in low risk obesity populations

Characteristics	7-day musculoskeletal pain			12-month musculoskeletal pain		
	No Pain	Pain	χ^2^	No Pain	Pain	χ^2^
Gender, n (%)						
Male	35(38.9)	55(61.1)	.227	26(27.7)	68(72.3)	.014
Female	37(35.6)	67(64.4)		28(26.9)	76(73.1)	
Age, n (%)						
18–44y	36(64.3)	20(35.7)		23(41.1)	33(58.9)	
45–64y	34(31.2)	75(68.8)	30.70**	29(25.9)	83(74.1)	11.91*
>64y	2(6.9)	27(93.1)		2(6.7)	28(93.3)	
Education, n (%)						
Informal	1(12.5)	77(87.5)		9(9.7)	84(90.3)	
Primary	11(39.3)	17(60.7)	48.71**	9(33.3)	18(66.7)	30.34**
Secondary	13(54.2)	11(45.8)		9(37.5)	15(62.5)	
Tertiary	37(68.5)	17(31.5)		27(50)	27(50)	
Comorbidity, n (%)						
No	60(53.6)	52(46.4)	30.75**	44(37.6)	73(62.4)	15.40**
Yes	12(14.6)	70(85.4)		10(12.3)	71(87.7)	
BMI, n (%)						
Underweight	1(25)	3(75)		1(25)	3(75)	
Normal	33(42.9)	44(57.1)	2.24	20(25.6)	58(74.4)	.177
Overweight/obese	36(32.7)	74(67.3)		32(28.3)	8(71.7)	
Physical activity, n (%)						
Low	12(14.5)	71(85.5)	32.07**	10(11.8)	75(82.2)	20.51**
Moderate	54(53.5)	47(46.5)		36(36.9)	65(63.1)	
High	6(60)	4(40)		6(60)	4(40)	

** associations significant at p<0.001

* association significant at p<0.05

In the last 7 days, logistic regression analysis showed that advancing age (odds ratio=1.09;95%CI:1.02–1.16) and comorbidity (odds ratio=3.0;95%CI:1.07–8.39) were risk factors associated with musculoskeletal pain, (χ^2^ (9) = 38.66, p<0.001) (Table 3). The model explained 33% variance in the risk of musculoskeletal pain (Nagelkerke, R^2^) and was able to identify 68% cases accurately. The sensitivity and specificity of the model were 71% and 65% respectively. The results showed that for every 1 unit increase in age and comorbidity status, the odds of occurrence of musculoskeletal pain in the last seven days increase by 1.09 and 3.00 times respectively. However, in the last 12 months, comorbidity and physical activity level had significant influence on musculoskeletal pain, (χ^2^ (9) = 32.77, p<0.001), at 43% sensitivity and 86% specificity. The results showed that comorbidity increased the odds of musculoskeletal pain in the last 12 months by 6 times (OR = 5.57, p = 0.006). However, adults with high physical activity level are 4% likely to develop musculoskeletal pain in the last 12 months.

**Table 3:** Results of binary logistic regression model on risk factors for musculoskeletal pain.

Characteristics	7-day musculoskeletal pain^1^		12-month musculoskeletal pain^2^	
	Odds Ratio (95% CI)	P Value	Odds Ratio (95% CI)	P Value
Age	1.09 (1.02–1.16)	**0.006**	1.04 (0.98–1.10)	0.23
Gender _Female_	0.80 (0.34–1.87)	0.60	0.43 (0.18–1.02)	0.06
Disease duration	1.01 (0.79–1.28)	0.96	0.91 (0.71–1.17)	0.47
Comorbidity	3.00 (1.07–8.39)	**0.04**	5.57 (1.62–19.17)	**0.006**
BMI _Normal_	0.67 (0.04–12.14)	0.78	1.93 (0.11–35.35)	0.66
BMI _overweight/obese_	0.80 (0.04–14.35)	0.89	1.18 (0.07–21.14)	0.91
Moderate _PA_	2.07 (0.74–5.81)	0.17	2.84 (0.93–8.69)	0.07
Vigorous _PA_	1.02 (0.28–3.75)	0.98	2.03 (0.54–7.63)	0.29
Total _PA_	0.33 (0.05–2.36)	0.27	0.06 (0.008–0.51)	**0.01**

1 Omnibus χ^2^ (9) = 38.66, p<0.001, R^2^ = 24.6% (Cox & Snell), 32.8% (Nagelkerke) Overall = 67.9% (SEN = 71.2%; specificity = 64.8%)

2 Omnibus χ^2^ (9) = 32.77, p<0.001, R^2^ = 21% (Cox & Snell), 29.1% (Nagelkerke) Overall = 71.2% (SEN = 42.6%; specificity = 85.9%)

## Discussion

This study found that the prevalence of musculoskeletal pain in adults with type 2 diabetes in populations with low risk of obesity was (72.7%) and proportionately similar between males and females. A similar prevalence of musculoskeletal pain among adults with type 2 diabetes has been observed in Europe (6). However, in the present study, the prevalence of musculoskeletal pain reported in the primary weight-bearing joints (lower back, knee and hip) seems lower compared to Molsted et al. with a different study population (Danish). In comparison, Molsted et al. collected history of pain in the last 14 days compared to the commonly used Nordic Musculoskeletal Questionnaire, which requested history of pain in the last seven days and 12 months. In addition, Molsted et al. was conducted among the Danish populations with a high prevalence of obesity (24). The presence of obesity increased the risk of lower limb osteoarthritis (25). Furthermore, our study population consisted of Hausa-Fulani residents, who are known to have slimmer stature and lower BMI (26). It is likely that a high proportion of them would have lower BMI, and hence prevalence of musculoskeletal pain in the lower body regions may be expected to be lower. Intuitively, the lack of association of musculoskeletal pain with obesity in our study may be partly due to reduced statistical power, resulting from collapsing of ‘overweight’ and ‘obese’ items into one item. Collapsing items for statistical convenience has previously been reported to be associated with loss of statistical information (27).

It has been observed that clustering of other components of metabolic syndromes (e.g. physical inactivity) increases the risk of diabetes-induced osteoarthritis (28). Furthermore, the lack of association thereof between obesity and increased prevalence of musculoskeletal pain appears not to be fixated on increased BMI; even adults with type 2 diabetes and normal weight but sedentary lifestyle may also be at risk. Unfortunately, the risk of musculoskeletal complications for adults with type 2 diabetes and obese compared to adults with type 2 diabetes and normal weight but who were inactive was not investigated in this study. However, in this study, physical activity and high level of education were strongly associated with a decreased risk of prevalence of musculoskeletal pain in patients with type 2 diabetes. Like in previous studies, participants with higher educational levels had lower risk of type 2 diabetes mellitus (29). Regular and increasing physical activity improve glycaemic control and reduce peripheral resistance to insulin (30), which hitherto played a protective role in reducing the formation of advanced glycation end products, a precursor of musculoskeletal complications in patients with type 2 diabetes (31). In line with the recommendations of van Dijk and colleagues (14), overwhelming evidence in the literature shows that exercise interventions, which include aerobic exercise, resistance exercise, flexibility training and balance training improve insulin sensitivity, endothelial function and glucose homeostasis in patients with type 2 diabetes. Public health education on the importance of physical activity should be undertaken to mitigate the effect of AGEs in musculoskeletal complications in patients with type 2 diabetes (32). Based on the differential estimates of sensitivity and specificity for our logistic regression models, it may be easier to associate age and comorbidity to musculoskeletal pain in the last seven days in adults with type 2 diabetes. However, poor sensitivity observed (43%) in participants who reported musculoskeletal pain in the last 12 months presents a difficult-to-diagnose condition in the long term. It is anticipated that sensitivity and specificity vary with disease spectrum and its prevalences (33,34). Overall, age and comorbidity may be more sensitive to acute musculoskeletal pain than a long-standing case of musculoskeletal pain in this population.

Nevertheless, this study had several limitations. First, the study had no control group, hence attributable risk for musculoskeletal pain in this population cannot be determined. Second, since this was a hospital-based study of the prevalence of musculoskeletal pain in adults with type 2 diabetes receiving care at selected hospitals, the prevalence of musculoskeletal pain in this population may be exaggerated by co-existence of other diseases. Third, the study may have been underpowered to detect the influence of BMI in the logistic regression model because we collapsed ‘overweight’ and ‘obese’ into one item. In conclusion, our results show that musculoskeletal pain is highly prevalent in adults with type 2 diabetes in populations with low risk of obesity. However, the pattern of the prevalence is lower in the primary weight-bearing joints. Advancing age together with comorbidity are associated with an increased risk of musculoskeletal pain in this population. The association of physical activity and tertiary educational level with the decreased odds of prevalence of musculoskeletal pain underscores the need to develop an evidence-based health education programme designed to promote physical activity in order to prevent and reduce the effect of diabetes-related musculoskeletal complications.

**Highlights**:

- Prevalence of musculoskeletal pain in adults with type 2 diabetes in populations with low risk of obesity is high and similar between men and women.
- Prevalence of musculoskeletal pain is higher in those who were older, having other medical conditions and sedentary.
- Advancing age and comorbidity are risk factors associated with musculoskeletal pain in adults with type 2 diabetes from a low risk obesity population.
